# Evaluation of mesenchymal stem cells’ bone repair capacity via osteogenic lineage differentiation in experimental nonunion fractures

**DOI:** 10.1186/s12891-026-10114-6

**Published:** 2026-06-18

**Authors:** Serhii Maslennikov, Mariia Isachenko, Maksym Danukalo, Olga Hancheva, Maksym Golovakha, Yurii Kolesnyk

**Affiliations:** 1 Department of Traumatology and Orthopedics, Educational and Scientific Institute of Postgraduate Education, Zaporizhzhia State Medical and Pharmaceutical University, Zaporizhzhia, Ukraine; 2 Department of Pathological Physiology with the course of Normal Physiology, Zaporizhzhia State Medical and Pharmaceutical University, Zaporizhzhia, Ukraine

**Keywords:** Mesenchymal stem cells, Osteogenic differentiation, Fracture nonunion, Bone regeneration, Rabbit model

## Abstract

**Background:**

Nonunion of fractures is a major challenge in orthopedics and traumatology, especially with increasing high-energy injuries. Adipose-derived mesenchymal stem cells (ASCs) are a readily accessible source with strong osteogenic potential. This study compared the bone regenerative efficacy of undifferentiated ASCs versus their osteogenically pre-differentiated derivatives in a critical-size femoral nonunion model.

**Methods:**

Eighteen male New Zealand White rabbits (*n* = 18), aged 3 months and weighing 2.94 ± 0.10 kg, were included. MSCs were isolated enzymatically from abdominal adipose tissue of three donor rabbits. At passage 3, cells were cultured either in basal medium (undifferentiated cohort) or osteogenic medium containing β-glycerophosphate, dexamethasone, and ascorbic acid for 4 weeks (differentiated cohort). 5-bromo-2′-deoxyuridine solution (BrdU) labeling was performed. In this randomized controlled in vivo study, a 5-mm mid-femoral defect was created and stabilized with angular-stable plating. In this randomized, controlled in vivo experimental study, on postoperative day 7 the animals were allocated to three groups (*n* = 5 per group): control (defect alone), undifferentiated mesenchymal stem cells, and differentiated mesenchymal stem cells. Radiographic assessment of bridging callus and cortical thickness index was performed at 4 and 6 weeks; serum total protein, calcium, and alkaline phosphatase were measured.

**Results:**

*In vitro*, osteogenic differentiation induced progressive mineralization (Alizarin Red staining) and a statistically significant 34% reduction in BrdU corrected total cell fluorescence (CTCF) (*p* = 0.0473). In vivo, both cell-augmented groups accelerated fracture consolidation versus controls.

The differentiated cohort achieved 88% bridging callus at 6 weeks (versus 80% in the undifferentiated group), with superior improvements in bridged cortices (+ 9.4%), bridging callus coverage (+ 10.0%), and distal gap closure (–31.0%) at 6 weeks, plus more pronounced cortical remodeling (average CTI decrease − 6.2% at 4 weeks). Serum alkaline phosphatase (ALP) declined by 53.4% (undifferentiated) and 63.6% (differentiated) versus control (both *p* < 0.001); plasma calcium was 16.8% higher in the undifferentiated group. Immunofluorescence showed 19% higher BrdU-positive cell density and 12% higher CTCF in undifferentiated cells.

**Conclusions:**

Osteogenically pre-differentiated ASCs demonstrated enhanced bone regenerative capacity compared with undifferentiated ASCs. These findings indicate that osteogenic pre-differentiation augments the therapeutic potential of ASCs and support their further evaluation in multimodal strategies for fracture nonunion.

## Background

Contemporary regenerative medicine is increasingly regarded as a promising avenue for the restoration of damaged tissues. Although fracture non-union is relatively uncommon, it remains one of the most challenging complications in orthopaedics and traumatology. Delayed union – defined as the absence of bone union within three months – is documented in approximately 600,000 fracture cases annually on a global scale [1]. International statistical data remain limited; nevertheless, the overall incidence of non-union is estimated to range from 5% to 10%, depending on the anatomical site of the fracture [2].

The effective management of such complications hinges on the integrated deployment of growth factors, osteoconductive scaffolds, cellular augmentation at the fracture site, and stable mechanical fixation within an optimised biological milieu – a strategy universally referred to as the “diamond concept” [3]. Within this framework, cellular therapies, above all the application of mesenchymal stem cells (MSCs), have shown marked therapeutic efficacy attributable to their self-renewal capacity, multilineage differentiation potential, and secretion of bioactive mediators that orchestrate regeneration of injured tissues [4]. Adipose tissue has gained recognition as a highly attractive alternative to bone marrow for MSC isolation, owing to its ready accessibility, substantially greater cellular yield, and virtual absence of ethical limitations [5]. Some authors posit that the osteogenic capacity of adipose-derived stem cells (ADSCs) may surpass that of bone marrow-derived MSCs, yet these observations still require independent confirmation [6]. In view of these benefits, the osteogenic differentiation potential of MSCs remains a central research priority in traumatology for the treatment of traumatic delayed union and nonunion.

Osteogenic differentiation of MSCs for bone repair represents a mature and intensively studied field. Bone marrow-derived MSCs (BM-MSCs) have been the subject of extensive investigation and are widely regarded as possessing superior osteogenic properties compared with ADSCs, which, despite easier procurement and higher cell numbers, generally exhibit lower osteogenic performance [7]. A large body of work has compared undifferentiated MSCs with their osteogenically pre-differentiated derivatives in diverse bone regeneration models. For example, in a rat cranial defect model, BM-MSCs promoted faster and more robust bone formation than ADSCs [8]. Likewise, an in vivo study utilising a shape-memory polymer scaffold demonstrated that osteogenically differentiated umbilical cord MSCs significantly enhanced new bone deposition relative to undifferentiated cells, thereby emphasising the advantages of pre-differentiation [9]. Nonetheless, both in vitro and in vivo comparisons reveal notable inconsistencies. While undifferentiated MSCs may support osteogenesis mainly through paracrine signalling (e.g., via osteoprotegerin), in vivo evidence consistently indicates superior direct bone matrix formation by differentiated MSCs [10]. In a canine radial fracture model, sheets of osteogenically differentiated MSCs outperformed their undifferentiated counterparts in promoting healing, suggesting that pre-differentiation can accelerate recovery [11]. Conversely, certain studies, including those in guinea pig fracture models, detected no significant outcome differences between differentiated and undifferentiated MSCs, although both exceeded control groups [12].

Despite extensive investigation, the impact of pre-differentiation on the mechanisms of tissue regeneration remains insufficiently understood [13–15]. It remains unclear whether undifferentiated MSCs, acting mainly through paracrine signaling and immunomodulation, or pre-differentiated cells, which directly contribute to matrix deposition, provide superior outcomes in clinical bone repair scenarios. The present in vitro and in vivo study addresses these critical gaps by providing a direct comparative analysis of the regenerative capacity of undifferentiated MSC cultures and their osteogenically differentiated derivatives in a clinically relevant model of nonunion fractures. By elucidating the differential contributions of differentiation status to cellular survival, engraftment, and long-term bone integration within a hostile injury microenvironment, the work offers new mechanistic insights with substantial potential to optimize cellular therapies. These findings may ultimately translate into more effective, standardized protocols for managing traumatic delayed union and nonunion, thereby reducing the socioeconomic burden of prolonged disability and repeated surgical interventions in orthopaedic traumatology.

The purpose of the study was to determine the specific features of the reparative influence exerted by mesenchymal stem cells and their differentiated lineage in an experimental model of fracture non-union.

## Methods

The study was approved by the Local Bioethics Committee of Zaporizhzhia State Medical and Pharmaceutical University (ZSMPhU) - Protocol No. 15, dated 10 December 2025. All stages of the study were conducted in the specially equipped Laboratory of Cell Cultures and Bioengineering of ZSMPhU, which forms a structural subdivision of the Educational and Scientific Medical-Laboratory Centre with Vivarium at Zaporizhzhia State Medical and Pharmaceutical University (Certificate of Technical Competence of the Ministry of Health of Ukraine No. 181/23, issued 21 December 2023 and valid until 20 December 2028), in full compliance with the highest standards of equipment quality, aseptic technique, sterility, and biosafety.

### Animals and study design

**Fig. 1:**
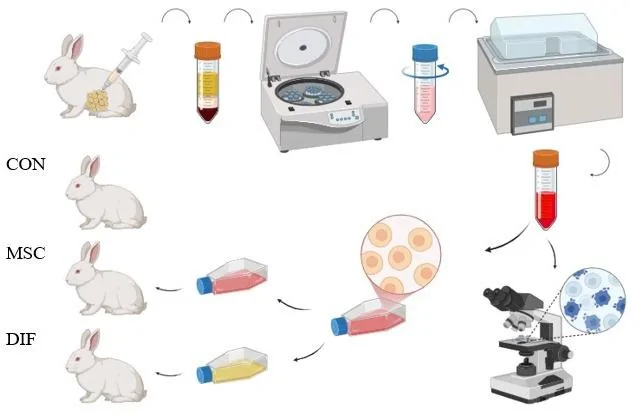
Study design

Eighteen male *New Zealand White* rabbits (*n* = 18), aged 3 months and weighing 2.94 ± 0.10 kg on average, were included in the study. Rabbits were selected as the experimental model because their femoral anatomy permits the reliable creation of a critical-size 5-mm segmental defect that does not heal spontaneously, thereby reproducing the clinical situation of fracture nonunion. The use of young adult New Zealand White rabbits ensures sufficient bone size for stable angular plating, standardized surgical access, and reproducible radiographic and histological assessment while maintaining acceptable ethical and economic parameters of the study. The animals were obtained directly by the research team of the Zaporizhzhia State Medical and Pharmaceutical University from a private farmer (Zaporizhzhia region, Ukraine) who provided written consent for their involvement in the research, followed by a quarantine period and the issuance of a veterinary certificate attesting to their health status. A primary mesenchymal stem cell line was established from three donor rabbits included in the present study. Adipose tissue was harvested directly by the research team from the subcutaneous (inguinal region) and visceral (perirenal and gonadal) depots under approved institutional ethical protocols. These adipose tissue depots served as the primary source of adipose-derived mesenchymal stem cells, which were isolated by enzymatic digestion and subsequently cultured under standard in vitro conditions [16]. The remaining 15 rabbits were randomly allocated to the three experimental groups (*n* = 5 per group) using a computer-generated randomisation sequence (simple randomisation) prepared by an independent researcher not involved in surgical procedures, postoperative care or outcome assessment. Allocation concealment was maintained until the moment of cell injection. Group 1 (control – CON) comprised control animals in which fracture non-union was induced; Group 2 (undifferentiated MSCs – MSC) consisted of animals that received an injection of MSCs directly into the fracture site; and Group 3 (osteogenically differentiated MSCs – DIF) involved the administration of MSCs that had undergone osteogenic differentiation (Fig. 1). The sample size of *n* = 5 animals per group was determined a priori based on power analysis derived from comparable rabbit femoral critical-size defect models reported in the literature [17]. Assuming a large effect size (Cohen’s f = 0.80) for the primary radiographic outcome (percentage of bridging callus), with a two-sided α = 0.05 and statistical power of 80%, the calculated minimum sample size per group was five animals. This number was retained in compliance with the 3R principles (Replacement, Reduction, Refinement) outlined in the *International Guiding Principles for Biomedical Research Involving Animals* (CIOMS, 1985) and the *Guide for the Care and Use of Laboratory Animals* (National Research Council, 2011). This approach aimed to minimize the number of animals used while allowing for preliminary histopathological evaluation and statistical comparisons in this exploratory study.

The operating surgeon was unaware of the specific cell type (undifferentiated versus osteogenically differentiated mesenchymal stem cells) contained in the syringe at the time of injection.

Nevertheless, radiographic evaluations, cortical thickness index measurements, immunofluorescence analyses, and biochemical assays were performed by two investigators blinded to treatment allocation to minimise observer bias.

### Isolation and culture of adipose-derived mesenchymal stem cells

The first stage of the study involved the isolation of mesenchymal stem cells from rabbit lipoaspirate by collagenase enzymatic digestion. Third-passage cells (P3), cultured for 15 days, were allocated to two distinct culture media in flasks (catalogue no. 690175, CELLSTAR^®^, Greiner Bio-One GmbH, Germany). In the first medium, the cells were expanded in high-glucose Dulbecco’s Modified Eagle Medium (DMEM; D6429, Sigma-Aldrich, Germany) supplemented with fetal bovine serum (FBS) and antibiotics; these cells were subsequently implanted into the fracture sites of rabbits in Group 2. In the second medium, the cells underwent directed osteogenic differentiation by incubation in low-glucose, pyruvate-containing, glutamine-free, phenol red-free DMEM (catalogue no. 11054020, Gibco, USA) supplemented with FBS, antibiotics, L-glutamine (A2916801, Gibco, USA), β-glycerophosphate disodium salt hydrate (G9422, Sigma-Aldrich, Germany), dexamethasone (D4902, Sigma-Aldrich, Germany), and L-ascorbic acid (A4403, Sigma-Aldrich, Germany); these differentiated cells were implanted into the fracture sites of rabbits in DIF-group.

The incubation of cells in both culture media was maintained for 4 weeks. At the end of each week, the efficiency of differentiation was visually assessed by cytochemical staining of the cultures with Carazzi’s haematoxylin and eosin, Alizarin Red (Morphisto, Germany), and Alcian Blue, in strict accordance with the manufacturers’ instructions. All microscopic evaluations performed at the stage described above were carried out on a ZEISS Primo Vert inverted microscope (Carl Zeiss, Germany) using an Axiocam 208 color digital camera.

Three days prior to the administration of the cell cultures to the rabbits, 5-bromo-2′-deoxyuridine solution (BrdU) ≥ 99% (HPLC) (catalogue no. B5002, Sigma-Aldrich, Germany) was added to the culture media. To confirm the efficiency of BrdU incorporation and the proliferative activity of the cultures, one of the control flasks was selected and the cells were transferred into an 8-well glass slide chamber (Millicell^®^ EZ Slide, catalogue no. PEZGS0816, Ireland).

Following a 2-day incubation period in the slide chamber, cells from both culture media were fixed in methanol and subsequently examined by indirect immunofluorescence using a mouse monoclonal anti-BrdU antibody (sc-32323, Santa Cruz Biotechnology, Inc., USA) and fluorescein isothiocyanate (FITC) - conjugated secondary antibodies (sc-2010, Santa Cruz Biotechnology, Inc., USA) according to the protocol described below. Before implantation, cell viability was assessed by the Trypan Blue exclusion test using a hemocytometer. Only preparations with viability exceeding 92% were used for in vivo administration. The average viability was 93.4 ± 1.8% for undifferentiated MSCs and 91.7 ± 2.3% for osteogenically differentiated MSCs, confirming high cell quality prior to transplantation.

### Surgical procedure and cell delivery

During the second stage of the study, the rabbits underwent surgical intervention to induce a model of fracture non-union. Because of the anatomical features of the skeleton and the specific objectives of this phase, the femur was selected as the target bone. All procedures were performed under sterile conditions in the operating theatre, with strict adherence to the principles of humane treatment of laboratory animals, as prescribed by the Law of Ukraine “On the Protection of Animals from Cruel Treatment” (No. 3447-IV of 21 February 2006) [18] and the European Convention for the Protection of Vertebrate Animals Used for Experimental and Other Scientific Purposes (Strasbourg, 18 March 1986) [19].

**Fig. 2:**
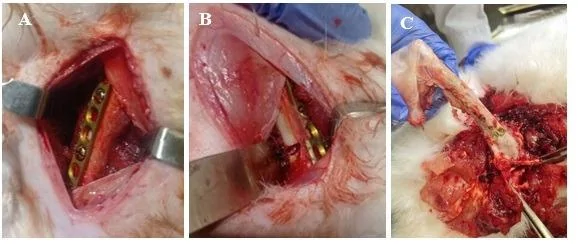
Fixation of the bone with an angular stability plate (**A**), the critical-size defect created (**B**), and preservation of fixator positioning at the time of withdrawal from the experiment (**C**)

For premedication, *meloxicam* solution was administered intramuscularly at a dose of 0.5 mg/kg 30` prior to surgery, followed 15` later by intraperitoneal injection of *Medison* solution (medetomidine hydrochloride) at 0.25 mg/kg. Anesthesia was induced by intramuscular administration of *Telazol* solution at 10 mg/kg, with continuous monitoring of vital parameters (respiratory rate and depth, heart rate, body temperature). Maintenance of anaesthesia was achieved by inhalation of *isoflurane* at 1–3% in 100% oxygen via a tight-fitting face mask (flow rate 0.5–1 L/min), titrated to effect according to reflexes and vital signs until the end of the short surgical procedure. The lateral aspect of the thigh was meticulously shaved and aseptically prepared with *Betadine* solution. A linear incision was performed over the middle third of the femoral shaft, followed by blunt muscle dissection and meticulous haemostasis. An angular stability plate was applied and fixed with four cortical screws. A 5-mm critical-size defect was then created between two adjacent screws using a drill bit. The surgical wound was thoroughly irrigated with antiseptic solutions, closed in layers, and covered with an aseptic dressing; the limb was immobilised in the extended position with an elastic bandage (Fig. 2).

To achieve complete reversal of the sedative, analgesic, and adverse cardiovascular and respiratory effects, and to facilitate gradual emergence from sedation, the specific antidote *Reverson* (atipamezole hydrochloride) was administered. In the postoperative period, *meloxicam* solution was employed as an anti-inflammatory and analgesic agent for the first 5 days.

An aseptic dressing was applied, and the limb was immobilized in an extended position with an elastic bandage for the first 5 postoperative days solely to prevent self-inflicted damage. No further immobilization was applied after day 5. From day 6 post-surgery, all animals were allowed full weight-bearing and unrestricted movement in their individual cages to simulate natural loading on the limb. Daily veterinary assessments confirmed no wound complications (infection, dehiscence, or inflammation) in any animal across all groups. All rabbits exhibited comparable full weight-bearing and normal cage activity from day 6 until the end of the study (6 weeks post-cell injection). This uniform ostoperative protocol ensured standardization of mechanical stimulation and recovery conditions across the control, MSCs, and Dif-MSCs groups.

On day 7 after operation, following sedation (*Medison* and its specific antidote *Reverson*), the rabbits received cell injections suspended in culture medium (1.2 × 10^7^ cells/ml in 1 ml culture medium) directly into the defect zone (percutaneous delivery route) under C-arm fluoroscopic guidance.

### Radiographic examinations

Subsequent radiographic examinations were performed on days 21 and 30 after cell administration. Evaluation of the regenerative process on radiographs was carried out using standard radiographic parameters in the ratologic mode: tissue thickness 2–4 cm, tube voltage 50–52 kV, and tube current-exposure time product 0.6–1.3 mAs. The animals were positioned supine with the operated limb abducted and internally rotated by three-quarters. The primary outcome measures were the percentage of bridging callus (% Bridging Callus), a quantitative indicator of callus formation as a critical stage in secondary fracture healing, and the cortical thickness index (CTI), employed for non-invasive dynamic monitoring of bone mineral density during the healing process.

Radiographic evaluation of bridging callus was performed according to a modified Lane and Sandhu scoring system [20]. The defect was assessed on orthogonal (anteroposterior and mediolateral) radiographs. Each of the four cortices was scored as bridged when continuous radiopaque callus connected the proximal and distal bone fragments. The percentage of bridging callus (% Bridging Callus) was calculated as the proportion of bridged cortices relative to the total number of cortices (number of bridged cortices / 4 × 100), with additional quantitative measurement of callus coverage along the defect length using ImageJ software. The CTI additionally served as a criterion for the absence of systemic bone pathology (e.g., osteoporosis) and as a supplementary marker of the condition of bone tissue within the fracture zone [21, 22].

Euthanasia of the rabbits was performed on day 45 following a 12-hour fast, using *Thiopental* (thiopental sodium 950.0 mg) at a dose of 120 mg/kg body weight. Once clear signs of cessation of vital functions were observed, blood was collected from the left cardiac ventricle, followed by excision of the femur.

### Laboratory research methods

The concentrations of total protein (g/L), calcium (mmol/L), and alkaline phosphatase activity (ALP, U/L) in blood were determined on a biochemical analyzer in accordance with the manufacturer’s instructions.

Following fixation of the bone specimens in 4% buffered formalin solution, the samples were meticulously cleared of adherent soft tissues and sectioned into 1 × 1 cm fragments, followed by thorough rinsing for 2 h under running cold tap water. Decalcification was subsequently performed over a period of 15 days in a 20% buffered solution of ethylenediaminetetraacetic acid (EDTA, pH 7.0–7.5). The completion of the decalcification process was verified using a mobile X-ray unit, X-MIND UNITY (Acteon Group, France).

The decalcified bone fragments were sequentially dehydrated in a graded series of ethanol solutions and cleared in mixtures of 100% ethanol with chloroform at ratios of 2:1, 1:1, and 1:2, for 2 h in each solution. The fragments were then cleared in pure chloroform and subsequently in a 1:3 mixture of chloroform and Paraplast (MkCormick, USA) at 37 °C. Finally, the specimens were embedded as paraffin blocks using the HistoStar tissue embedding station (Thermo Fisher Scientific, USA).

For histochemical techniques, serial Sect.  5 μm thick were prepared from the histologically processed fragments of decalcified bone using a Microm HM325 rotary microtome (Thermo Fisher Scientific, USA). The resulting sections were mounted on adhesive microscope slides (SuperFrost Menzel-Gläser, 75 × 25 × 1.0 mm, Germany) and dried in a Red Line thermostat (Binder, Germany) for 7–10 days.

For immunofluorescence studies, the protocol supplied by the manufacturer, Santa Cruz Biotechnology, Inc. (USA), was employed. Following the standard histochemical processing described above, tissue antigens were retrieved by heat-induced epitope retrieval in 10 mM sodium citrate buffer, pH 6.0 (Santa Cruz Biotechnology, Inc., USA, catalogue no. sc-294091) using a PT-module (Thermo Scientific, USA) for 20 min at + 95 °C. After cooling to room temperature, the slides were immersed in phosphate-buffered saline (PBS) and washed in three changes of buffer for 5 min each. Excess fluid was aspirated under dark-room conditions, and 300 µL of UltraCruz^®^ blocking reagent (Santa Cruz Biotechnology, Inc., USA, catalogue no. sc-516214) was applied per slide. Primary antibodies directed against BrdU (as detailed above) were diluted directly in this blocking solution at a working concentration of 1:64. The slides were subsequently washed three times in PBS. For indirect immunofluorescence, the primary antibodies were applied, and the slides were incubated for 24 h at + 4 °C. After three additional washes in PBS, the sections were incubated for 1 h in a humidified chamber at + 37 °C with FITC-conjugated secondary antibodies. For direct immunofluorescence, antibodies already conjugated to FITC were used. Following final washing, the sections were mounted in glycerol suitable for fluorescence microscopy (catalogue no. 1040950250, Sigma-Aldrich, Germany) or in UltraCruz mounting medium containing 4′,6-diamidino-2-phenylindole (DAPI) (Santa Cruz Biotechnology, Inc., USA, catalogue no. sc-24941), according to the specific requirements of each experiment.

All fluorescence microscopy examinations, performed at both the in vitro and in vivo stages, were conducted on an AxioImager M2 microscope (Carl Zeiss, Germany) using 38HE high-emission filter sets (Carl Zeiss, Germany) (green fluorescence: λex = 470/40 nm, λem = 525/50 nm for the markers; blue fluorescence: λex = 360 nm, λem = 460 nm for DAPI) and a high-sensitivity video camera, AxioCam ERc 5s (Carl Zeiss, Germany). Digital image analysis was carried out with the open-source software ImageJ (National Institutes of Health, USA).

The following parameters were quantified during immunofluorescence analysis of both cultured cells and rabbit bone sections: the number of DAPI-positive and BrdU-positive cells per microscopic field, the BrdU-positive area per field, the BrdU-positive cell density (cells/mm²), and the corrected total cell fluorescence (CTCF) of BrdU. Each microscopic field measured 150,185 μm².

Pathohistological processing was performed using a classical protocol. Staining was carried out using hematoxylin-eosin (H&E) and Van Gieson methods. Photomicrographs were analyzed using the open-source software ImageJ (National Institutes of Health, USA). Cells were identified morphologically using standard histological criteria without immunostaining: osteoblasts as cuboidal cells with hyperchromatic nuclei along bone surfaces and osteocytes as embedded cells in lacunae within the bone matrix. Counts were performed manually in a blinded manner across 10 high-power fields (HPF, ×400) per section, with at least three sections per animal (totaling 30 HPF per sample).

Cell numbers were expressed as absolute counts per 10 HPF and averaged across sections. Quantitative analysis of osteoblasts and osteocytes was performed exclusively in the callus regeneration zone of specimens obtained 6 weeks after cell injection.

Statistical analysis was performed using one-way analysis of variance (ANOVA) in the Statistica software package (licence No. JPZ804I382130ARCN10-J). Continuous variables are presented as median (1st and 3rd quartiles). For comparison of two independent groups, an unpaired two-tailed Student’s t-test was used. For comparison among three independent groups one-way analysis of variance (ANOVA) was applied, followed, in the case of statistical significance, by Tukey’s post hoc test for multiple comparisons. A two-sided p-value of less than 0.05 was considered statistically significant for all tests.

## Results

### In vitro

On the first day of MSCs culture, robust adhesion was observed. The adherent cells exhibited a heterogeneous morphological profile. Multipolar and spindle-shaped cells were readily distinguishable. Subsequently, as the cells proliferated and their density within the monolayer increased, the cell morphology underwent progressive changes, characterized by the formation of polar protrusions from which cytoplasmic processes extended.

On day 13, the cells were first passaged. Microscopic evaluation revealed an uneven cellular distribution throughout the flask, accompanied by the formation of distinct proliferative foci. At this stage, the culture consisted predominantly of spindle-shaped and polygonal cells displaying prominent filopodia, with rounded cells being virtually absent.

Fourteen days after the first passage, cytological preparations stained with hematoxylin and eosin demonstrated that the cells retained the phenotypic features acquired during proliferation.

**Fig. 3:**
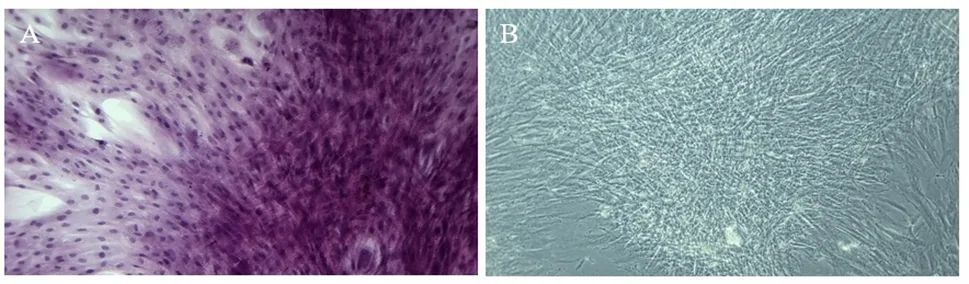
Mesenchymal stem cells (passage 1) on day 28 of culture. **A** Hematoxylin and eosin staining, original magnification ×10. **B** Phase-contrast microscopy (ZEISS Primo Vert), original magnification ×4

Polygonal cells with prominent filopodia remained evident, as did spindle-shaped cells bearing short cytoplasmic processes. Notably, pronounced cytoplasmic vacuolization and large nuclei were observed (Fig. 3).

**Fig. 4:**
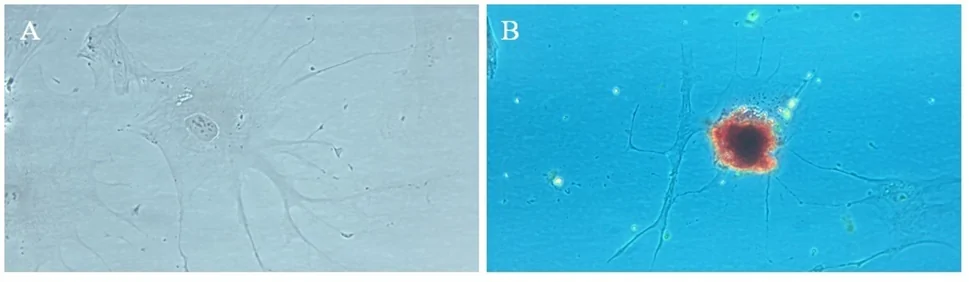
Calcium deposition in mesenchymal stem cells demonstrated by Alizarin Red staining on day 30 of culture. **A** Undifferentiated control MSCs. **B** Osteogenically differentiated MSCs. Original magnification ×40 (ZEISS Primo Vert microscope)

After induction of osteogenic differentiation, only small, scattered calcium deposits – possibly artifactual or baseline in nature – were observed in control MSCs on day 8. In contrast, Alizarin Red staining in the osteogenic medium was markedly more extensive and diffuse, covering a substantially greater area and likely representing an early stage of mineralized extracellular matrix formation (Fig. 4).

On days 24 and 30 of culture, control MSC samples remained morphologically unaltered and exhibited no signs of calcification. In contrast, osteogenically differentiated cultures displayed large, intensely stained, round red foci, indicating clear progression of extracellular matrix calcification. Differentiation was validated by a 3.8-fold increase in ALP activity (spectrophotometric assay at 405 nm, *p* < 0.001 vs. undifferentiated MSCs) and positive Alizarin Red S staining, with quantified calcium deposition covering 48.6% ± 9.2% of the culture area (image analysis via ImageJ, *p* < 0.001 vs. 1.8% ± 0.4% in undifferentiated controls). No spontaneous differentiation was observed in undifferentiated MSCs, confirming distinct cell states prior to implantation.

Immunofluorescence staining for BrdU, with nuclear counterstaining using DAPI, confirmed efficient incorporation of the label into cells of both undifferentiated MSCs and osteogenically differentiated MSCs (Table 1).

**Table 1:** BrdU labeling indices in mesenchymal stem cell cultures determined by immunofluorescence. Data are presented as median (Q1; Q3)

Indicators		MSC	DIF	*p*-values were calculated using unpaired two-tailed Student’s t-test
Cell number:	BrdU^+^	3.00 (2.00; 4.00)	3.00 (3.00; 4.75)	*р*= 0.7907
	DAPI^+^	8.50 (6.25; 11.50)	8.00 (7.25; 10.75)	*р*= 0.9527
Cell density BrdU^+^/mm^2^		13.32 (8.35; 21.06)	13.40 (13.34; 16.70)	*р*= 0.8714
Cell area BrdU^+^		4271.07 (2837.88; 5081.41)	3291.24 (2218.56; 4845.33)	*р*= 0.2980
CTCF BrdU^+^		127400.37 (92931.07; 217256.39)	84200.17 (45456.72; 160133.00)	***р*** **= 0.0473**

Bold values indicate statistically significant differences (*p* < 0.05)

Cells from both experimental groups retained high viability and proliferative capacity following isolation and expansion. This was evidenced by efficient BrdU incorporation into DNA, as shown by the presence of BrdU⁺/DAPI⁺ cells, indicating active DNA synthesis at the time of labeling.

A statistically significant 34.0% decrease in corrected total cell fluorescence (CTCF) was observed in osteogenically differentiated MSCs compared with undifferentiated controls (*p* = 0.0473), which is consistent with successful induction of osteogenic differentiation via cell cycle withdrawal and G0/G1 arrest.

### In vivo

Biochemical analysis of plasma samples from rabbits in the experimental groups showed no statistically significant differences in total protein concentration. This observation indicates the maintenance of systemic protein homeostasis regardless of the intervention performed (Table 2).

**Table 2:** Plasma biochemical parameters in rabbits of the experimental groups. Data are presented as median (Q1; Q3)

Indicators	Control	MSC	DIF	*p*-values were calculated using one-way analysis of variance (ANOVA) followed by Tukey’s post hoc test for multiple comparisons
Total protein, g/l	74.6 (72.9; 87.4)	71.3 (69.1; 77.8)	70.8 (68.2; 78.5)	p*= 0.1783 p**= 0.1788 p***= 0.9577
Calcium (Ca), mmol/l	2.39 (2.35; 2.57)	2.79 (2.72; 2.88)	2.54 (2.41; 2.59)	***p*** *= 0.0010 p**= 0.4136 ***p*** ***= 0.0008
Alkaline phosphatase activity, U/L	418.7 (385.4; 472.1)	195.2 (181.6; 242.9)	152.3 (131.7; 177.8)	***p*** *= 0.0001 ***p*** **= 0.0001 p***= **0.0145**

Bold values indicate statistically significant differences (*p* < 0.05)

p* - Control vs. MSC, p** - Control vs. DIF, p*** - MSC vs. DIF

Plasma calcium concentrations were significantly elevated in the MSC group compared with controls (by 16.8%; two out of three intergroup comparisons, *p* = 0.0010 and *p* = 0.0008), and remained 9% higher than in the osteogenically differentiated group. These data indicate that undifferentiated MSCs stimulate systemic calcium mobilization to facilitate bone regeneration, whereas differentiated cells primarily exert local effects, with minimal impact on circulating levels – presumably because a substantial fraction of calcium is already incorporated into the mineralized matrix within the culture.

Alkaline phosphatase activity was substantially and statistically significantly decreased in both MSC (-53.4%) and differentiated (-63.6%) groups relative to controls (all three intergroup comparisons reached statistical significance, *p* ≤ 0.0145). Although a decrease in ALP alone cannot be interpreted as definitive evidence of superior healing, this reduction is consistent with accelerated transition from the reparative to the remodeling phase of fracture healing, as previously documented in both experimental and clinical studies of nonunion management [23].

**Fig. 5:**
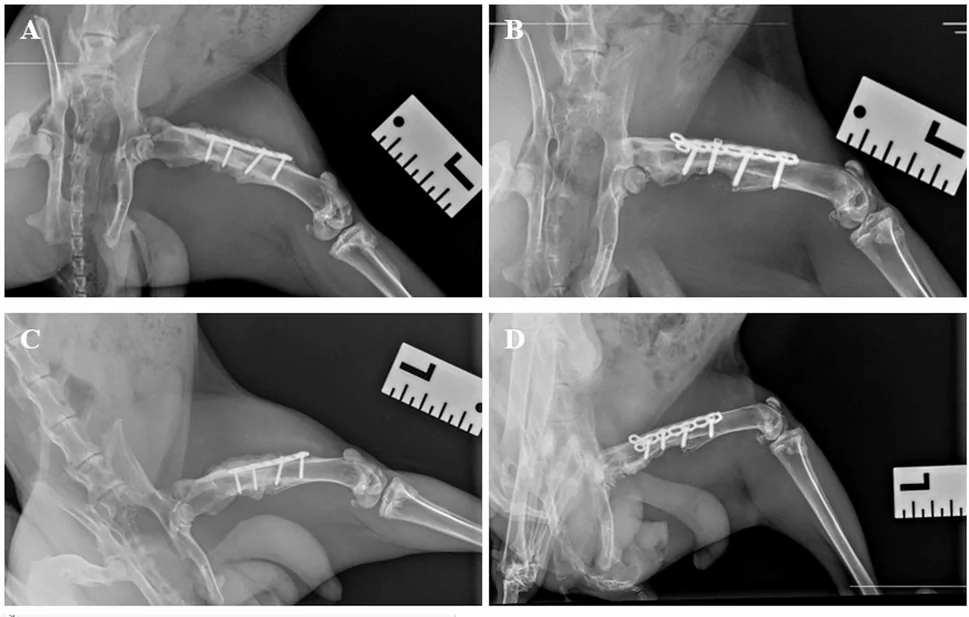
Representative radiographs of the operated hind limbs in experimental rabbits. **A**, **B** 4 weeks after cell transplantation: (**A**) MSC group, (**B**) osteogenically differentiated MSC group. **C**, **D** 6 weeks after cell transplantation: (**C**) MSC group, (**D**) osteogenically differentiated MSC group

Radiographic assessment of the experimental rabbits demonstrated the following changes (Fig. 5; Table 3).

**Table 3:** Radiographic parameters in the MSC group at 4 and 6 weeks after cell transplantation into the fracture site. Data are presented as median (Q1; Q3)

Indicators	MSC		DIF		*p*-values were calculated using one-way analysis of variance (ANOVA) followed by Tukey’s post hoc test for multiple comparisons
	4 weeks	6 weeks	4 weeks.	6 weeks	
Bridged Cortices, %	2.29 (2.23;2.31)	3.20 (3.14;3.21)	2.70 (2.65;2.70)	3.50 (3.45;3.50)	***p*** *=0.0001 ***p*** **=0.0001 ***p*** **#=0.0001** ***p*** **##=0.0001**
Bridging Callus, %	57.30 (55.80;57.70)	80.00 (78.50;80.20)	68.00 (67.80;68.20)	88.00 (87.80;88.00)	***p*** *=0.0001 ***p*** **=0.0001 ***p*** **#=0.0001** ***p*** **##=0.0001**
Distal Gap, %	39.10 (39.10;43.20)	17.40 (16.20;17.80)	33.00 (32.50;33.80)	12.00 (12.00;12.50)	***p*** *=0.0001 ***p*** **=0.0001 ***p*** **#=0.0001** ***p*** **##=0.0001**
CTI Distal (Cortical thickness index)	0.49 (0.49;0.50)	0.49 (0.47;0.49)	0.47 (0.46;0.47)	0.48 (0.47;0.48)	p*=0.1328 p**=0.0955 ***p*** **#=0.0068** *p*##=0.5346
CTI Proximal	0.46 (0.45;0.46)	0.44 (0.44;0.44)	0.43 (0.42;0.43)	0.44 (0.43;0.44)	p*=0.1434 p**=0.0955 ***p*** **#=0.0085** *p*##=0.3734
CTI Average	0.48 (0.47;0.48)	0.46 (0.45;0.46)	0.45 (0.44;0.45)	0.46 (0.45;0.46)	***p*** *=0.0216 p**=0.0955 ***p*** **#=0.0022** *p*##=1.0000
D, mm (Total external diameter at the fracture site (bone + callus))	11.90 (11.70;12.00)	11.00 (10.90;11.10)	10.50 (10.30;10.60)	10.40 (10.30;10.40)	***p*** *=0.0048 p**=0.4492 ***p*** **#=0.0002** ***p*** **##=0.0021**
d, mm (Internal medullary canal diameter at the fracture site)	5.70 (5.70;6.20)	6.50 (6.50;6.60)	5.50 (5.40;5.50)	5.60 (5.50;5.60)	***p*** *=0.0221 p**=0.2029 ***p*** **#=0.0304** ***p*** **##=0.00001**

Bold values indicate statistically significant differences (*p* < 0.05)

p* - MSC group, 4 weeks vs. 6 weeks; p** - osteogenically differentiated MSC group, 4 weeks vs. 6 weeks; *p*# - MSC group vs. DIF group at 4 weeks; *p*## - MSC group vs. osteogenically differentiated MSC group at 6 weeks

Radiographic assessment of rabbits from both experimental groups revealed unequivocal signs of fracture consolidation and gap closure. This was evidenced by statistically significant improvements in Bridged Cortices, Bridging Callus, and Distal Gap scores at week 6 versus week 4 (multiple *p*-values < 0.05 across time points, Table 3). Of note, the proportion of bridged cortical layers and the percentage of fracture zone covered by callus were significantly greater in the osteogenically differentiated MSC group at both time points (all four intergroup comparisons *p* < 0.05) : 17.9% and 18.7% higher at week 4, and 9.4% and 10.0% higher at week 6, respectively. These data indicate more rapid cortical consolidation under the influence of differentiated cells, most likely attributable to their targeted osteogenic activity, as opposed to the broader, non-specific effects of undifferentiated MSCs. 

Osteogenically differentiated cells appear to promote faster structural union by circumventing certain intermediate stages of in vivo differentiation. The observed pattern also demonstrates enhanced callus formation in the differentiated group, likely resulting from localized cellular transdifferentiation and optimization of the reparative process. This strategy may represent a superior regenerative approach, particularly in clinical settings involving complex fractures where rapid healing is paramount.

Furthermore, the incidence of a persistent distal gap was markedly lower in the differentiated group compared with the MSC group at both time points (-15.6% at week 4 and − 31.0% at week 6), confirming more effective gap closure.

Of particular interest was the divergent bone remodeling pattern observed at week 4 in the osteogenically differentiated MSC group compared with the undifferentiated MSC group, as indicated by the CTI Distal, CTI Proximal, and CTI Average values. Differentiated cells elicited a more pronounced and accelerated phase of remodeling and resorption, reflected in statistically significant decreases in CTI Distal (4.1%), CTI Proximal (6.5%), and CTI Average (6.2%). This reduction is likely associated with soft callus formation and the initiation of endochondral ossification, driven by localized osteoclast activation. Thus, the administration of osteogenically differentiated MSCs appears to expedite the remodeling process by more rapidly recruiting and activating osteoclasts, leading to transient cortical thinning in both proximal and distal regions. By week 6, however, this effect was offset by secondary osteogenesis and increased bone density. The contrasting distal and proximal CTI profiles further suggest that differentiated cells selectively enhance proximal bone density, most likely as a result of uneven cell distribution at the injection site or differential biomechanical loading of the operated limb (Fig. 5; Table 3).

Differences in the effects produced by undifferentiated MSCs and osteogenically differentiated MSCs were also apparent in bone geometric parameters, specifically the external diameter at the fracture/callous site and the internal diameter of the medullary canal at the fracture site. Injection of osteogenically differentiated MSCs led to the formation of a less voluminous but more organized and remodeled callus by week 4, as evidenced by a statistically significant 11.8% reduction compared with the MSC group. This advantage persisted at week 6. By contrast, at week 6 the medullary canal in the MSC group was significantly narrower (more occluded) than in the differentiated group (by 13.8%), indicating divergent remodeling patterns or distinct priorities between the two cell types.

Immunofluorescence analysis of bone tissue from control rabbits demonstrated the complete absence of BrdU-specific immunoreactivity (Table 4).

**Table 4:** BrdU immunofluorescence labeling indices in bone tissue of experimental rabbits. Data are presented as median (Q1; Q3)

Indicators	Control	MSC	DIF	*p*-values were calculated using one-way analysis of variance (ANOVA) followed by Tukey’s post hoc test for multiple comparisons
Cell density DAPI^+^/ mm^2^	315.47 (216.88; 368.04)	460.05 (407.48; 532.35)	378.35 (350.90; 446.91)	***р*** *= 0.0007 ***р*** **= 0.0039 ***р*** ***= 0.0400
Cell density BrdU^+^/mm^2^	0.01 (0.01; 0.01)	129.59 (108.78; 156.16)	105.16 (83.87; 105.16)	***р*** *= 0.0000 ***р*** **= 0.0000 ***р*** ***= 0.0398
Cell area BrdU^+^	0.01 (0.01; 0.01)	20.24 (12.76; 25.96)	20.47 (15.30; 28.49)	***р*** *= 0.0000 ***р*** **= 0.0000 р***= 0.5439
CTCF BrdU^+^	160.14 (108.31; 296.31)	1462.26 (1084.11; 2874.99)	1284.80 (1222.41; 2043.72)	***р*** *= 0.0000 ***р*** **= 0.0000 ***р*** ***= 0.0435

Bold values indicate statistically significant differences (*p* < 0.05)

p* - Control vs. MSC, p** - Control vs. DIF, p*** - MSC vs. DIF

**Fig. 6:**
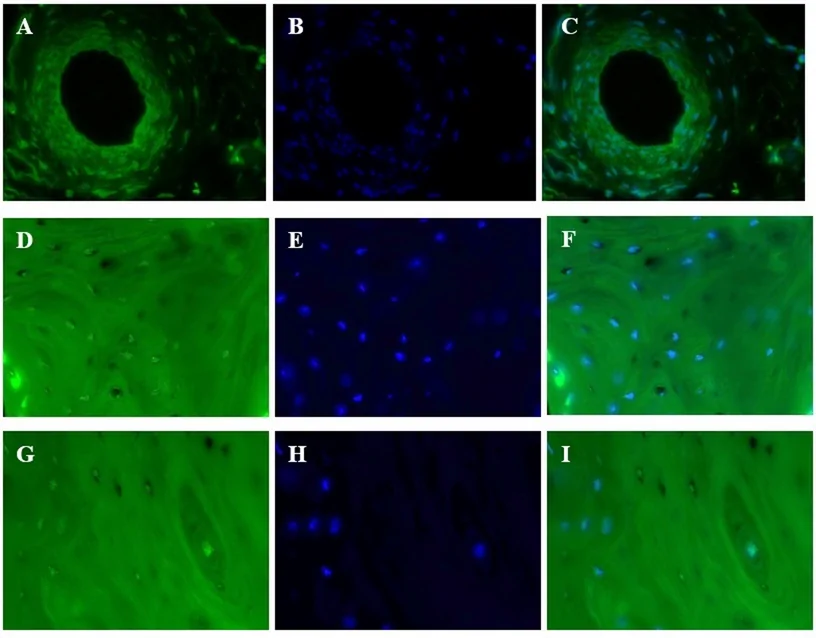
BrdU⁺ cells (double-labeled with DAPI) in bone tissue of experimental rabbits. Representative images acquired with a Zeiss Axio Imager.M2 microscope. **A**-**F** MSC group, original magnification ×40. **G**-**I** Osteogenically differentiated MSC group, original magnification ×40

Immunofluorescence micrographs of bone tissue from experimental rabbits revealed substantial differences in the proliferative activity of bone cells between animals that had received either undifferentiated MSCs or osteogenically differentiated MSCs (11 out of 12 intergroup comparisons, *p* ranging from < 0.0001 to 0.0435; Table 4). In the MSC group, BrdU⁺ cells were not restricted to the fracture site but displayed a diffuse distribution across remote bone regions. This distribution was associated with a statistically significant 19% increase in the density of labeled cells compared with the differentiated MSC group (*p* = 0.0398), while the area occupied by BrdU⁺ cells remained unchanged (Fig. 6; Table 4).

The CTCF index exhibited changes analogous to those recorded in cell culture experiments.

Specifically, a statistically significant 12% increase was detected in the MSC group relative to rabbits in the DIF cohort (*p* = 0.0435). Consequently, DIF cells proved to be more discretely localized and to possess lower proliferative activity, a finding fully consistent with their differentiated cellular status.

Histomorphometric and quantitative cellular evaluation of bone regeneration, reported in detail in the companion manuscript, confirmed the radiographic findings. The number of osteoblasts was significantly higher in the undifferentiated MSC group (52.0 ± 16.2 per 10 high-power fields) compared with the control group (28.6 ± 7.0, *p* = 0.032). In the osteogenically differentiated MSC group osteoblast counts averaged 40.2 ± 10.5 per 10 HPF. Osteocyte density showed significant intergroup variability (ANOVA *p* = 0.014) and was highest in the Dif-MSC group (68.7 ± 14.3 per 10 HPF) compared with the MSC group (45.3 ± 5.62, *p* = 0.006). These data demonstrate a clear regenerative hierarchy (Control < MSC < Dif-MSC) and indicate that undifferentiated MSCs primarily stimulate osteoblast proliferation, whereas osteogenically pre-differentiated cells promote further maturation into osteocytes and formation of organised lamellar bone.

## Discussion

The results obtained in the present experimental study provide compelling evidence of the pronounced reparative potential of mesenchymal stem cells isolated from adipose tissue, as well as their osteogenically predifferentiated derivatives, in a model of femoral fracture nonunion.

Administration of DIF was associated with accelerated and more complete fracture consolidation, as corroborated by a composite of radiological, biochemical, and immunohistochemical parameters. In particular, a statistically significant increase in Bridging Callus, reduction of the distal interfragmentary gap, and optimization of bone remodeling processes collectively indicate more effective restoration of the mechanical integrity of the bone.

The observed reduction in alkaline phosphatase activity within the DIF group, coupled with a decrease in cellular proliferative activity as evidenced by BrdU labeling, is consistent with a more mature and spatially confined pattern of osteogenesis. Immunofluorescence analysis demonstrated robust long-term cell survival in both cohorts up to 6 weeks post-transplantation. Undifferentiated MSCs exhibited higher BrdU-positive cell density and a more diffuse distribution throughout the bone, reflecting greater migratory capacity, whereas osteogenically predifferentiated cells showed more discrete localization at the defect site with lower proliferative indices. This pattern indicates superior retention and functional integration of predifferentiated cells within the injury microenvironment. Furthermore, the absence of clinical or histological signs of inflammation or immune rejection despite the allogeneic nature of the transplanted cells highlights the potent immunomodulatory properties of adipose-derived mesenchymal stem cells, which likely created a favourable local milieu by suppressing excessive inflammatory responses and promoting tolerance.

This finding is consistent with the principle of controlled regeneration that avoids excessive cellular expansion. In this context, the results align closely with the “Diamond Concept” of nonunion management, which posits the synergistic interplay of a cellular component, adequate mechanical stability, and an osteoconductive microenvironment [24]. A review of contemporary literature corroborates the rationale for employing adipose-derived mesenchymal stem cells as a viable alternative to bone marrow-derived cells. *Mott A.et al.* (2020) [25] demonstrated that the application of MSCs in the treatment of nonunions and bone defects promotes osteogenesis in 70–90% of cases, particularly when combined with biomaterials or autologous bone grafts. Furthermore, adipose-derived mesenchymal stem cells are distinguished by substantially superior accessibility, markedly less invasive harvesting, and a higher stem cell yield per unit volume compared with their bone marrow-derived counterparts, although the optimization of their osteogenic differentiation remains an active and unresolved area of investigation. Systematic analyses of experimental studies conducted by *Saputra A. et al.* (2025) [26] indicate that osteogenically induced adipose-derived MSCs exhibit upregulated expression of key bone matrix proteins, most notably type I collagen and osteocalcin. These findings position such cells as highly promising candidates for the regenerative management of fracture nonunions. In the context of cell therapy, particular attention is drawn to the question of the appropriateness of predifferentiating mesenchymal stem cells (MSCs) prior to transplantation. Our results, which demonstrate the superior efficacy of DIF, are consistent with the hypothesis that osteogenic predifferentiation reduces the requirement for cells to undergo the full differentiation cascade in vivo. This, in turn, shortens the time to formation of mineralized matrix and lowers the risk of undesired differentiation along alternative lineages [27]. In previous experimental models, predifferentiated adipose-derived MSCs have been shown to exhibit upregulated expression of the transcription factors Runx2 and Osterix and to display enhanced integration into the zone of bone defect. Similar mechanisms likely underlie the reduction in the number of BrdU-positive cells recorded in the DIF group in the present study, which may reflect the cells’ exit from the cell cycle and their functional specialization toward synthesis of bone matrix.

The radiological and biochemical alterations identified in the present study demonstrate clear parallels with findings from other experimental investigations. In particular, animal fracture models have shown that enhanced vascularization and activation of vascular endothelial growth factor (VEGF) -mediated pathways are accompanied by an increase in callus volume of up to 80–88% and a concomitant reduction in inflammatory markers, thereby facilitating more effective regeneration [23].

The integration of these effects with local MSC administration, as implemented in our model of a 5-mm atrophic defect, may exert an additional synergistic action. Likewise, it has been demonstrated that the application of adipose-derived MSCs in combination with biopolymer carriers accelerates the healing of nonunions, elevating bone mineral density by 15–20% within 6 weeks [1], a finding that correlates closely with our CTI values and the observed reduction in Distal Gap.

Clinical reviews addressing the application of mesenchymal stem cells (MSCs) in the management of delayed union and nonunions underscore their capacity to stimulate both osteogenesis and angiogenesis via the secretion of growth factors, most notably VEGF and bone morphogenetic protein-2 (BMP-2). This process results in enhanced vascularization of the fracture zone and the establishment of a conducive microenvironment for regeneration [28]. According to Kiwan E. et al*.* [29] local delivery of MSCs achieves high union rates; however, the authors stress the critical need to regulate cellular differentiation potential in order to avert adipogenic transdifferentiation. In this respect, our findings of a less pronounced systemic calcium mobilization in the DIF group provide further evidence in favour of the more targeted and safer local action of predifferentiated cells. Data from clinical studies in humans corroborate the therapeutic promise of cell-based approaches for the management of nonunions. Although the majority of existing clinical protocols are predicated on the use of bone marrow-derived mesenchymal stem cells, a growing body of authors highlights the distinct advantages of predifferentiated MSCs, which are attributable to their superior quantitative yield and markedly reduced age-related functional decline. Additionally, Theodosaki A. M. et al. [30] demonstrated that the combination of MSCs with scaffolds enhances the efficacy of nonunion treatment by 20–30% relative to traditional methods, as confirmed by validated scales of fracture healing assessment [9]. In this context, the osteogenic differentiation protocol employed in the present study, which utilizes β-glycerophosphate, dexamethasone, and ascorbic acid, faithfully replicates the approaches adopted in clinically oriented protocols and thereby accounts for the observed enhancement of calcification and cellular integration. The reduction in alkaline phosphatase activity documented in the cell therapy groups is consistent with findings from clinical investigations conducted within the framework of the “Diamond Concept,” in which normalization of this marker is regarded as indicative of the transition from the phase of active osteoblastic matrix synthesis to the remodeling stage, accompanied by a reduction in chronic inflammation.

Although the present findings demonstrate robust long-term cell survival and a favourable immune profile up to 6 weeks after transplantation, several important questions regarding clinical scalability remain to be elucidated. These results open promising perspectives for the development of allogeneic “off-the-shelf” mesenchymal stem cell products; however, they also highlight the need for further investigation into large-scale manufacturing under Good Manufacturing Practice (GMP) conditions, optimization of standardized cryopreservation protocols, and the establishment of rigorous potency assays. Future translational studies should therefore address these issues by evaluating the cells in larger animal models that incorporate clinically relevant comorbidities, by incorporating advanced biomaterial-based delivery platforms (such as scaffolds or hydrogels), and ultimately by conducting carefully designed human clinical trials to determine optimal dosing regimens, timing of administration, and the most effective integration of osteogenically predifferentiated adipose-derived MSCs into multimodal regenerative strategies.

### Study limitations

The present study has several limitations inherent to its experimental design. First, the relatively small sample size (*n* = 5 per group), although determined a priori by power analysis and fully compliant with the 3R principles, restricts statistical power and precludes robust subgroup analyses or detection of smaller effect sizes. Second, although BrdU labeling provided valuable information on cell survival and distribution, the study design could not fully discriminate between direct cellular contribution to osteogenesis and paracrine/immunomodulatory effects. These limitations notwithstanding, the present work offers a mechanistically focused comparative analysis of undifferentiated versus osteogenically pre-differentiated adipose-derived mesenchymal stem cells in a clinically relevant nonunion model and provides a solid foundation for subsequent larger-scale and translational investigations.

## Conclusions

In conclusion, the present study demonstrates that the administration of adipose-derived mesenchymal stem cells, particularly those predifferentiated along the osteogenic lineage, promotes accelerated consolidation of experimental femoral non-union fractures. It ensures the most rapid and qualitatively superior formation of bone callus together with restoration of structural integrity at the fracture site, promotes the efficient completion of the phase of intense remodeling and metabolic reorganization, and simultaneously minimizes systemic calcium mobilization compared with untreated controls. Osteogenically predifferentiated cells were associated with more advanced radiographic outcomes in terms of bridging callus formation, gap closure, and bone remodeling, while also showing reduced systemic calcium mobilization. Immunofluorescence analysis of BrdU-positive cells constitutes an effective method for delineating cellular phenotypes. It allows confirmation of the status of mesenchymal stem cells as actively proliferating and migrating progenitor cells, while characterizing osteogenically differentiated cells as localized predifferentiated osteogenic material labeling proved to be a useful tool for tracking cell survival and distribution, revealing distinct patterns of localization and proliferative activity between undifferentiated and differentiated cell populations.

Collectively, these findings indicate that osteogenically predifferentiation may enhance the bone regenerative capacity of adipose-derived MSCs in a model of fracture nonunion. The results support the potential of predifferentiated ASCs as a promising component in cell-based strategies for the management of fracture nonunions. Further preclinical and clinical studies are warranted to validate these observations and to explore their integration into multimodal regenerative approaches based on the Diamond Concept. 

## Data Availability

The datasets used and/or analysed during the current study are available from the corresponding author on reasonable request.

## Abbreviations

ALP: Alkaline phosphatase
ASCs: Adipose–derived mesenchymal stem cells
BMP–2: Bone morphogenetic protein–2
BrdU: 5–bromo–2′–deoxyuridine
CTCF: Corrected total cell fluorescence
CTI: Cortical thickness index
DAPI: 4′,6–diamidino–2–phenylindole
DMEM: Dulbecco’s Modified Eagle Medium
EDTA: Ethylenediaminetetraacetic acid
FBS: Fetal bovine serum
MSC(s): Mesenchymal stem cell(s)
PBS: Phosphate–buffered saline
VEGF: Vascular endothelial growth factor
